# Compositional Consequences of Ultrafiltration Treatment of White and Red Wines

**DOI:** 10.3390/foods13121850

**Published:** 2024-06-13

**Authors:** Stephanie Angela, David Wollan, Richard Muhlack, Keren Bindon, Kerry Wilkinson

**Affiliations:** 1 Discipline of Wine Science and Waite Research Institute, The University of Adelaide, PMB 1, Glen Osmond, SA 5064, Australia; stephanie.angela@adelaide.edu.au (S.A.); richard.muhlack@adelaide.edu.au (R.M.); 2 The Australian Research Council Training Centre for Innovative Wine Production, PMB 1, Glen Osmond, SA 5064, Australia; david.wollan@gmail.com; 3 VAF Memstar, P.O. Box 794, Nuriootpa, SA 5355, Australia; 4 The Australian Wine Research Institute, P.O. Box 197, Glen Osmond, SA 5064, Australia; keren.bindon@awri.com.au

**Keywords:** membranes, phenolics, polysaccharides, proteins, ultrafiltration, wine

## Abstract

Clarification and stabilisation processes are routinely performed post-fermentation to ‘finish’ wines, but traditional methods are slow and energy intensive, create waste, and can affect wine volume and quality. New methods that ‘finish’ wine rapidly, with higher recovery rates, and reduced waste and input costs, are therefore needed. Ultrafiltration is a separation process that fractionates liquids, nominally, according to molecular weight. By comparing the composition of permeate and retentate derived from pilot-scale fractionation of white and red wine using 75, 20, or 10 kDa membranes and different degrees of permeation (50, 80, 90, or 95%), this study sought to evaluate ultrafiltration as an innovative approach to the clarification and stabilisation of wine. Mass balance analysis confirmed that titratable acidity and alcohol were fractionated according to the degree of permeation; however, proteins, polysaccharides, and phenolic compounds (including anthocyanins for red wine) were concentrated in retentate due both to the membrane molecular weight cut-off (MWCO) specifications and degree of permeation. The retention of wine constituents smaller than the nominal MWCO suggests that interaction with other macromolecules or the membrane surface occurred. Red wine permeates were stripped of much of their essential character and were no longer considered commercially acceptable. In contrast, the removal of protein and phenolic compounds from white wine demonstrated the potential for ultrafiltration to remediate heat unstable or excessively phenolic wines. Findings enabled the identification of other winemaking applications of ultrafiltration technology that could enhance wine quality, process efficiency, and profitability.

## 1. Introduction

Ultrafiltration (UF) is a pressure-driven, membrane-based separation process that fractionates liquids, nominally, on the basis of molecular weight [1]. Depending on the molecular weight cut-off (MWCO) specifications of a membrane, larger molecules will typically be concentrated in the retentate, while smaller molecules pass into the permeate. However, it is important to recognise that the membrane material (inorganic, polymeric, or mixed matrix) and configuration [1], feed composition and flow rate, and applied pressure [2,3] also influence separation efficiency, such that MWCO alone does not predict the compositional outcomes of UF [3,4].

UF membranes have been used to fractionate and/or concentrate macromolecules of interest in a range of pharmaceutical, biotechnology, food and beverage, wastewater, and plant extraction applications [1,5,6]. Within food and beverage production, UF technologies are routinely used to concentrate milk proteins for cheesemaking [7,8,9], clarify and pasteurise fruit and vegetable juices [8,10,11,12], and recover protein from effluent derived from processing fish and poultry [8,13]. To date, however, there are limited wine-related applications of UF published in the scientific literature, albeit other membrane filtration processes (i.e., using microfiltration (MF), nanofiltration, and reverse osmosis) have been reported [14,15,16,17,18].

In winemaking, clarification and stabilisation processes are routinely performed post-fermentation to ‘finish’ wines; i.e., to remove constituents that increase the risk of undesirable physical or sensory changes occurring between bottling and consumption [19]. Haze-forming proteins are removed via the addition of bentonite (a clay-based colloid that readily binds proteins) to achieve protein stabilisation. Bentonite and other fining agents are also used to remove constituents that adversely affect wine sensory properties, including phenolic compounds responsible for astringency (harshness), bitterness, or browning [19]. Cross-flow MF and UF have been used for wine clarification [14,20,21], but the retention of proteins, phenolic compounds, and polysaccharides by UF membranes (due to smaller pore diameters [14] and, thus, lower nominal MWCO specifications), could enable the adoption of UF as an alternative approach to other stabilisation processes.

Several studies have evaluated the potential for UF to remove haze-forming proteins from grape juice or wine, either directly [4] or in combination with heat and protease treatments [22,23], as alternate approaches to heat stabilisation (than traditional bentonite fining [24]). Although stabilisation was not fully achieved due to permeation of some heat-unstable protein, the bentonite requirement (relative to unfiltered juice or wine) was substantially reduced, affording financial and environmental benefits. Ultrafiltration and nanofiltration have been used in combination to lower the sugar content of grape must (as a strategy for managing wine alcohol content), via a process patented by Bucher Vaslin as REDUX^®^ [25,26]. Using this process, UF first concentrates macromolecules (anthocyanins, polyphenols, polysaccharides, and proteins) in retentate, before nanofiltration of the resulting permeate is performed to concentrate sugars. The permeate obtained from nanofiltration, largely comprising water and acids, can then be blended with the UF retentate, yielding treated must comprising a lower sugar concentration that can be used to make wine of a lower alcohol content. A more recent study evaluated the potential for industrial-scale UF treatment of white and rosé wines to remove phenolic compounds associated with bitterness and astringency [27]. UF treatments achieved >90% fractionation of wines, with macromolecules being concentrated in retentates, such that permeates were not only heat stable, but had decreased brown colour and phenolic compounds. Other compositional parameters (pH, free and total sulphur dioxide, volatile acidity, and viscosity) were not significantly affected, and, so, changes in permeate composition were moderate compared with those of retentate; i.e., permeates were similar to their corresponding wine, but had decreased protein and phenolic compounds [27].

Membrane filtration offers several advantages over traditional winemaking processes, namely: technical efficiency by combining clarification, stabilisation and sterile filtration in a continuous operation; cost savings through reduced wine loss, energy consumption, and use of additives; and environmental benefits by reducing waste from the use of fining agents such as bentonite and activated carbon [26]. The results from recent protein stabilisation and phenolic management applications of UF to white wines are promising [22,23]. Nevertheless, further research into the compositional consequences of UF of wine is needed to fully realise the potential applications of this technology to winemaking.

This study investigated the effects of (i) the nominal membrane MWCO (being the lowest molecular weight at which 90% of a solute with a known molecular weight is retained by the membrane, albeit different membrane manufacturers use different methods for qualifying MWCO specifications) and (ii) the degree of permeation on the composition of permeate and retentate derived from the UF fractionation of white and red wine. The key objectives were to evaluate the use of UF for the management of phenolic compounds (specifically those associated with astringency or bitterness) and proteins (associated with haze formation) in white wine, as well as potential uses for the macromolecule-enriched retentate. Nevertheless, UF was also applied to red wine, despite the anticipated removal of anthocyanins and tannins (associated with colour and mouthfeel) being inherently detrimental to red wine quality, so as to fully characterise the chemical consequences of UF.

## 2. Materials and Methods

### 2.1. Wine Samples

Ultrafiltration (UF) was performed on three wines: a 2020 white wine (Sauvignon Blanc) sourced from Pernod Ricard Winemakers (Rowland Flat, SA, Australia); a 2019 red wine (comprising several red grape cultivars) sourced from the University of Adelaide winery (Urrbrae, SA, Australia); and a high-phenolic and oxidised wine made by submerging Sauvignon Blanc grape marc (~20 kg, portioned into four grain mash bags) in the aforementioned white wine (~60 L) for three months in an 80 L plastic drum (i.e., with ullage), stored in a 10 °C coldroom. Following extraction/oxidation and to protect the UF system, the high-phenolic wine was pre-filtered using a 10 inch filter cartridge with a nominal pore size of 1 micron (Kegland, Noble Park North, VIC, Australia), to remove any residual suspended solids (as a result of the grape marc treatment) prior to membrane filtration.

### 2.2. Ultrafiltration of White and Red Wines

**Figure 1:**
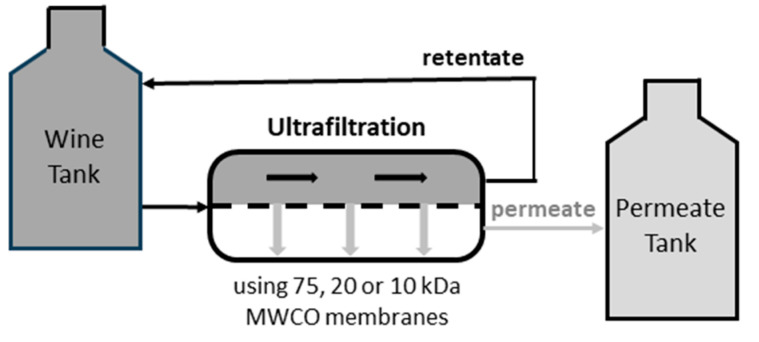
Schematic of the ultrafiltration system.

Ultrafiltration treatment of the white and red wines was performed (in duplicate) using a pilot-scale Micro AA bench top crossflow filtration system (VAF Memstar, Nuriootpa, SA, Australia), according to the manufacturer’s standard operating procedures. The system (Figure 1) was equipped with: spiral-wound polyethersulfone UF membranes (supplied by VAF Memstar), with nominal molecular weight cut-off specifications of 75, 20 or 10 kDa (being representative of the membranes being used to treat wine); a pressure pump (CMG, Rowville, Vic., Australia); and a heat exchange coil (to maintain wine temperatures at ≤20 °C). Wine was pumped from the feed tank and across the UF membrane (at constant pressure, i.e., 10 bar) to generate permeate and retentate. The permeate was continuously captured in a collection tank, while the retentate was cooled before being circulated back to the feed tank (Figure 1). Retentate and permeate samples were collected after 50, 80, 90, and 95% fractionation of the white wine, and after 50, 80, and 90% fractionation of the red wine; noting that attempts at 95% fractionation of the red wine resulted in blockage of the membrane. Different volumes of wine were fractionated to ensure sufficient retentate for compositional analyses, with the fractionation endpoint calculated as the volume of permeate collected relative to the initial feed volume (Table 1). On completion of each UF treatment, a warm alkaline solution (2% aqueous sodium hydroxide, buffered to pH < 12, at 40 °C) was circulated through the system, followed by neutralisation (with 3% aqueous citric acid) and then rinsing with water (as specified by the manufacturer).

### 2.3. Ultrafiltration of the Highly Phenolic Wine

UF treatment of the highly phenolic wine was performed (in triplicate) as outlined above but using only the 10 kDa membrane and a fractionation endpoint of 95% based on the conditions previously determined to be optimal for UF treatment of white wine.

### 2.4. Compositional Analysis of Wine, Retentate and Permeate

The physico-chemical composition of wine, retentate, and permeate samples were determined to establish the partitioning of different wine constituents as a function of both membrane nominal MWCO and the degree of permeation.

#### 2.4.1. Basic Chemistry

Wine pH and titratable acidity (TA) were determined by using an autotitrator (T50 model, Mettler Toledo, Port Melbourne, Vic., Australia). Wine alcohol content was determined using a DMA 4500 M Alcolyser (Anton Paar, Graz, Austria). Conductivity was measured using an ST300C-B conductivity meter (OHAUS, Port Melbourne, Vic., Australia). Free and total sulfur dioxide were measured by the Australian Wine Research Institute’s (AWRI) Commercial Services Laboratory (Adelaide, SA, Australia) using a Gallery Discrete Analyser (Thermo Fisher Scientific, Adelaide, SA, Australia) [28].

#### 2.4.2. Wine Colour, Tannins, and Phenolics

Red wine colour parameters were determined using the modified Somers colour test in Axygen^®^ 1.1 mL sealed 96 deep well plates (Thermo Fisher Scientific, Adelaide, SA Australia). A buffer containing 0.5% tartaric acid in 12% aqueous ethanol (pH 3.4) was prepared (buffer 1). Three measures were taken following dilution of wine (one in 10, with buffer 1 alone, buffer 1 containing 0.375% *w*/*v* sodium metabisulphite or with 0.1% *v*/*v* acetaldehyde), thorough mixing, and incubation for 1 h at room temperature. For a fourth measure, wine samples were also diluted (one in 50, with 1 M HCl), mixed, and incubated for 3 h at room temperature in the dark. Samples were then transferred to a UV-Star Greiner Bio-one GmbH 96-well plate (Interpath, Somerton, VIC, Australia) and measured at 280, 420, and 520 nm in a FLUOstar Omega microplate reader (BGM Labtech, Mornington, VIC, Australia), with path-length correction. Wine colour parameters were determined using the calculations outlined by [29]. Wine tannin concentration was determined by the high-throughput methyl cellulose precipitable tannin (MCPT) assay [29]. White wine samples were diluted (one in 10, with 1 M HCl) and then transferred to a UV-Star Greiner Bio-one GmbH 96-well plate and phenolics and brown colour intensity measured at 280 nm and 420 nm absorbance, respectively, using a Cary 60 UV-Vis spectrophotometer (Agilent, Mulgrave, VIC, Australia).

#### 2.4.3. Polysaccharides

Wine polysaccharide analysis was conducted according to previously published methodology [30]. Briefly, a 1 mL aliquot of wine was precipitated in 5 mL of absolute ethanol at 4 °C for 18 h. Precipitated samples were centrifuged at 3267× *g* for 20 min and washed twice with 1 mL ice-cold 80% ethanol solution. The remaining supernatant was discarded, and the dried pellets were reconstituted in 1 mL Milli-Q water and freeze-dried for 18 h. Lyophilised samples were resuspended in 300 µL of Milli-Q water, and 100 µL of the sample was combined with 100 µL of 4 M TFA and hydrolysed at 100 °C for 3 h. Hydrolysates were cooled on ice, and thereafter dried for 18 h using a Christ rotational vacuum concentrator (RVC-2-25 CDplus, Scitek, NSW, Australia), before being resuspended in 300 µL of Milli-Q water. A 30 µL aliquot of internal standard was added to 30 µL of each sample to give a final concentration of 0.3 M of ribose and deoxy-glucose (Sigma Aldrich, St. Louis, MO, USA). The derivatisation agent used was 0.5 M of methanolic 1-phenyl-3-methyl-5-pyrazolone (PMP) (Sigma Aldrich) in 1 M NH_4_OH. For the derivatisation step, 25 µL of sample containing internal standard was mixed with 96.2 µL of derivatising reagent and heated at 70 °C for 1 h before being cooled on ice and neutralised with formic acid. The samples were then extracted twice with dibutyl ether (Sigma-Aldrich), the supernatant was extracted manually, with the remaining dibutyl ether removed under vacuum for 20 min at room temperature. The PMP-monosaccharide derivatives were then quantified by HPLC using a C18 column (Kinetex, 2.6 μm, 100 Å, 100 × 3.0 mm) with an in-line filter (KrudKatcher Ultra HPLC in-line filter, 2.0 μm; Phenomenex, Lane Cove, NSW, Australia). The mobile phase was solvent A, 10% *v*/*v* 40 mM aqueous ammonium acetate, and solvent B, 70% *v*/*v* acetonitrile in Milli-Q water. HPLC method used was: 92% solvent A at 0 min; 84% at 12 min; 0% at 12.5 min, then returning to starting conditions (92% solvent A) from 14.5 to 18.5 min. A flow rate of 0.6 mL/min was used with a column temperature of 30 °C. The PMP-monosaccharide derivatives were identified using commercial monosaccharide standards (Sigma Aldrich).

#### 2.4.4. Proteins

White wine proteins (i.e., chitinases and thaumatin-like proteins) were quantified by HPLC using a previously published method [31]. A 2 mL aliquot of sample was filtered (0.45 μL PVDF syringe filter, Grace, Columbia, MD, USA) and 15 μL injected into an Agilent 1260 UHPLC and quantified against a commercial thaumatin standard (Sigma Aldrich).

### 2.5. Statistical and Mass Balance Analysis

Compositional data were subjected to analysis of variance (ANOVA) using GenStat (23rd edition, VSN International, Hemel Hempsted, UK), with one- and two-way ANOVA applied to data for the white and red wine, retentate, and permeate samples, and one-way ANOVA applied to data for the high-phenolic wine, retentate, and permeate samples. Mean comparisons of treatments were performed by Tukey-HSD post-hoc test at a significance level of *p* = 0.05. Mass balance analysis was also performed on a subset of compositional data to estimate the proportion of acids, alcohol, protein, phenolics, anthocyanins, and polysaccharides that were either retained by the membrane, passed into the permeate, or removed due to membrane adsorption, fouling, or precipitation. This was achieved by calculating the concentration of each constituent in retentate and permeate fractions, as a percentage of their initial concentration in wine.

## 3. Results and Discussion

### 3.1. Composition of Retentate and Permeate following UF of White Wine

White wine was fractionated by UF treatment using 20 or 10 kDa MWCO membranes, with retentate and permeate samples collected for compositional analysis following 50, 80, 90, or 95% permeation, to determine the partitioning of key constituents (Table 2), including protein, phenolic compounds, and polysaccharides.

Following UF, small increases in retentate and permeate pH were observed (0.16–0.23), relative to the pH of the initial wine. This was consistent with the ±0.2 changes in pH reported following industrial-scale fractionation of white and rosé wines by UF [27], and likely reflects electrostatic interactions with the membrane [32]. The pH of retentate and permeate varied by less than 0.05 and 0.04, respectively, and whilst statistically significant differences in pH were observed between corresponding retentate and permeate fractions, they were ≤0.06 and would, therefore, be unlikely to meaningfully affect wine sensory properties (i.e., the perception of acidity).

In contrast, more substantial differences in TA were observed between corresponding retentate and permeate samples. TA was consistently higher in retentate than in permeate, and, following 95% permeation (irrespective of membrane MWCO), was also significantly higher than in the initial wine. This suggests that some organic acids were retained by the membrane, despite having molecular weights being considerably lower than the nominal MWCO of the UF membranes, in agreement with previous research [27]. Again, this likely reflects electrostatic and/or adsorption interactions with the membrane. Mass balance calculations indicated that the TA content of retentate closely reflected the degree of fractionation (Table S1), whereas permeate TA was consistently lower than expected (by 4–5% and 3–9% for the 20 and 10 kDa MWCO membranes, respectively). As a consequence, only ~96–97% of TA was accounted for following UF treatment with the 20 kDa membrane. The tighter 10 kDa membrane may have resulted in stronger solute/membrane (and/or solute/solute) interactions, such that only 88–93% of TA was accounted for following 50 to 90% permeation. However, 98% of TA was accounted for after 95% permeation with this membrane. The concentration of organic acids and their salts (e.g., anionic tartrates, and potassium and calcium cations) in retentate explains the observed increase in its associated conductivity (being a measure of ionic strength [33]), while in permeate, changes in conductivity were small (≤0.06 ms/cm).

The alcohol concentration of retentates was consistently higher than that of their corresponding permeates (Table 2), in contrast to the significant decreases in retentate alcohol content reported following the industrial-scale UF fractionation of white and rosé wines reported previously (after ≥90% permeation) [27], but in agreement with outcomes from pilot-scale (~20 L) UF treatment of white wines (after 80% permeation) [23]. Relatively small differences in alcohol content (±0.2 abv, relative to the initial wine alcohol content) were observed following 95% fractionation, irrespective of membrane MWCO, but more substantial variation was observed with other UF treatments. Changes in retentate alcohol content were again consistent with the degree of fractionation, based on mass balance calculations (Table S1). Mass balance calculations accounted for ~97–99% of alcohol following UF with the 20 kDa MWCO membrane, but only 88–93% of alcohol following 50 to 90% fractionation using the 10 kDa MWCO membrane. Whereas dilution explained the decreased retentate ethanol concentrations observed in the aforementioned industrial-scale UF study (due to the use of water to recover retentate from the membrane filtration system [27]), this was unlikely in the current study, given that 98% of alcohol was accounted for after 95% permeation with the 10 kDa MWCO membrane. Presumably, this instead reflects some adsorption of ethanol by the 10 kDa MWCO membrane; however, this was not evaluated further in the current study.

The most dramatic compositional changes in response to UF were observed for wine macromolecule concentrations (Table 2). Following 50% fractionation by UF, the protein, total phenolics, and polysaccharide content of retentate either decreased slightly (due to solute/membrane and/or solute/solute interactions and fouling [34,35]) or remained the same, relative to initial wine concentrations. However, these constituents were then progressively concentrated in retentate, as a function of both the membrane MWCO and the degree of permeation. Following 95% fractionation, ~3- to 6-fold increases in protein, phenolic, and polysaccharide concentrations were observed, while the tighter 10 kDa membrane yielded ~4- to 10-fold increases in concentration, presumably due to greater retention of larger macromolecules by the narrower membrane pores. Mass balance calculations confirmed the preferential retention of these constituents in retentates, especially after ≥80% fractionation, and with the 10 kDa membrane (Table S1). However, the mass balance assessment also indicated that some loss of macromolecules had occurred for protein and polysaccharides in particular. Only 43–84% of protein and 61–84% of polysaccharides were accounted for when the mass balance calculations were considered. While 100 and 94% of total phenolics were accounted for following 95% permeation with the 20 and 10 kDa membranes, respectively, only 50% of phenolics were accounted for following UF treatment with the 10 kDa membrane at lower permeation rates. The loss of macromolecules might be attributable to fouling, i.e., either direct adsorption by the membrane or deposits that formed by accumulation on the membrane surface or due to colloidal instability post-filtration [35,36].

There were no significant differences amongst permeate protein concentrations, regardless of membrane MWCO or permeation rates, indicating there was passage of small quantities of protein throughout UF treatment. The molecular mass of wine proteins ranges from 9 to 88 kDa [37], with the most abundant (pathogenesis-related) proteins responsible for haze-formation, primarily thaumatin-like proteins (TLPs) and chitinases, having molecular masses of ~15–30 kDa [38]. The majority of these proteins, nominally, 90%, were expected to be rejected by UF membranes (especially the 10 kDa membrane). As such, some of the protein observed in permeates might reflect smaller lipid transfer proteins [39], or increased passage due to progressive concentration of protein in retentate. Whereas many of the wines subjected to industrial-scale UF treatment yielded heat-stable permeate due to complete removal of protein [27], one permeate retained 6.8 mg/L of protein (after 97.8% fractionation of a white wine comprising 18.9 mg/L of protein, with a 5 kDa MWCO membrane), i.e., a comparable outcome to that achieved in the current study.

Similar polysaccharide concentrations were observed in permeate samples derived from UF, but lower concentrations were generally observed following fractionation with the tighter 10 kDa MWCO membrane. In the case of phenolic compounds, small but statistically significant differences were observed amongst the permeate samples, suggesting there was passage of low molecular weight phenolic compounds (and/or other constituents that contribute to absorbance at 280 nm) throughout UF.

It is worth noting that variation in the fractionation outcomes of the different UF treatments may also reflect solute/solute interactions in the retentate, especially as wine macromolecules become concentrated in the progressively decreasing volume of retentate volume, and subsequently interact with the membrane or membrane deposits.

### 3.2. Composition of Retentate and Permeate following UF of Red Wine

**Figure 2:**
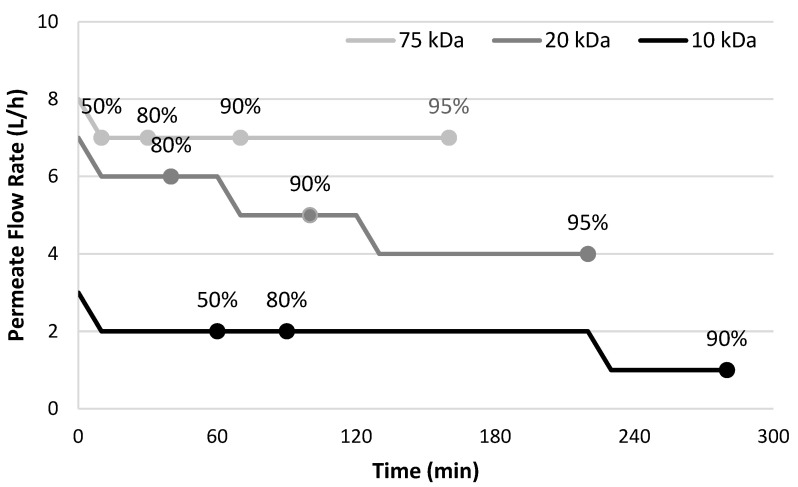
Permeate flow rate (L/h) during pilot-scale ultrafiltration treatment of red wine with 75, 20, or 10 kDa MWCO membranes, to different degrees of permeation (50, 80, 90, and 95%); flow rates were identical between duplicate treatments (i.e., standard deviation is zero).

Red wine was fractionated by UF treatment using 75, 20, or 10 kDa MWCO membranes, and again sought to achieve 50, 80, 90, and 95% permeation. However, whereas permeate flow rates of 6–8 L/h were maintained during UF of white wine), comparable flow rates were only obtained for red wine using the 75 kDa MWCO membrane (Figure 2). The permeate flow rate progressively declined (from 7 to 4 L/h) during UF fractionation of the red wine with the 20 kDa membrane, presumably due to fouling attributable to polysaccharides and phenolics [34,35] exacerbated by concentration polarisation, but 95% permeation was still achieved. In contrast, UF using the tighter 10 kDa membrane resulted in flow rates of just 2 L/h, and prior to 90% permeation flow rate decreased to 1 L/h, such that UF beyond 90% permeation was not possible. Compositional analysis of wine, retentate, and permeate samples was performed to determine the partitioning of key red wine constituents (Table 3), including anthocyanins, total phenolics, tannins, and polysaccharides.

The increase in pH observed following UF of white wine (Table 2) did not occur with UF treatment of red wine, such that significant pH differences were only observed following UF with the 10 kDa MWCO membrane. In this case, retentate pH decreased by 0.1 (after 80 or 90% permeation), while permeate pH increased by 0.06–0.07 (in the corresponding samples). The abundance of phenolic compounds inherently present in red wine (typically ~10-fold higher concentrations than for white wine [40]) may have resulted in their preferential interaction with either the membrane or other solutes, relative to organic acids. Nevertheless, UF of red wine had a similar impact on TA as that observed for UF of white wine. TA was again consistently higher in retentate than in permeate and was also significantly higher than that of the initial wine following ≥80% permeation (or ≥50% permeation using the 10 kDa MWCO membrane). This suggests that organic acids were again retained by the UF membranes, even by the 75 kDa membrane. Mass balance calculations indicated that retentate TA was consistently higher than expected based on the degree of fractionation (Table S2). For UF with 75 and 20 kDa membranes, 94–96% and 91–96% of TA was accounted for, respectively, whereas, following UF with the 10 kDa membrane, only 83% was accounted for after 90% fractionation. This suggests that a partial loss of organic acids occurred, such that their loss may have been exacerbated by the increased fouling that occurred with the tighter membrane, as evidenced by the lower flow rate (Figure 2).

The initial conductivity of the red wine was higher than that observed for the white wine, reflecting greater ionic strength. Following UF of red wine, significantly higher conductivity values were obtained in retentate after either 95% permeation with 75 or 20 kDa membranes or ≥50% permeation with the 10 kDa membrane. As a consequence, the permeate conductivity was significantly lower than the corresponding retentate, particularly after UF with the 10 kDa membrane, but differences were not significant by the degree of permeation. Again, changes in conductivity likely reflect variation in the retention of salts present in wine by the different membranes.

No significant differences were observed between the alcohol concentrations of corresponding retentate and permeate samples, irrespective of the degree of permeation. Mass balance calculations indicated changes in alcohol content were generally within 3% of the expected concentrations based on fractionation and accounted for 95–99% of the alcohol content of the initial red wine (and 98–99% after ≥90% permeation). It was, therefore, evident that UF had a minimal effect on the alcohol content.

Again, the greatest compositional differences arising from the UF of red wine were observed for wine macromolecules (Table 3). Comparisons between retentate and permeate composition indicated that anthocyanins, phenolic compounds, tannins, and polysaccharides were substantially rejected by the UF membranes, such that they were increasingly concentrated in retentate (as a function of both the degree of fractionation and the membrane MWCO). However, mass balance calculations indicated that a significant proportion of these macromolecules were also removed (Table S2), having presumably been retained by the membrane itself due to adsorption, fouling, and/or precipitation.

Anthocyanins are the principal source of red wine colour [41], along with oligomeric pigments (proanthocyanidins) and red polymeric pigments (tannins) [42,43]. The molecular weight of most anthocyanins ranges from ~450 to 670 Da [41], so based on size exclusion principles alone, anthocyanins would be expected to permeate the UF membranes used in the current study. Instead, anthocyanins were predominantly retained during UF treatments. As a consequence, their concentrations and wine colour density significantly increased in retentate. Greater than 4-fold concentration of anthocyanins was observed following 95% permeation with 75 and 20 kDa membranes, or 90% fractionation with the tighter 10 kDa membrane. This was supported by mass balance calculations, which also suggested that a loss of anthocyanins between 35 and 55% losses of anthocyanins occurred with ≥90% fractionation by UF (Table S2). Following UF treatment with the 10 kDa membrane, permeate samples comprised just 1–2% of the anthocyanins that were present in the initial wine, and thus, they were severely depleted of colour (Figure S1). Given that colour provides an important first cue of red wine quality for consumers, this was not a commercially acceptable outcome for red wine but could afford blending components for white or rosé wine.

Beyond 80% fractionation, a significant increase in the total phenolics content of retentate was observed, relative to both the initial wine and to corresponding permeate samples. Permeate derived from UF with the 75 and 20 kDa membranes accounted for total phenolics concentrations that were ≤35% of that observed in the initial wine (Table S2), whereas ≤4% of phenolic compounds permeated the 10 kDa membrane. Again, differences in the total phenolic content of permeate samples were observed according to membrane MWCO, but not the degree of permeation. Tannins were not detected in any of the permeate samples, suggesting that their higher molecular weight (relative to monomeric phenolics), and/or colloidal effects, resulted in their complete rejection by the UF membranes.

Only trace levels (i.e., 0.03–0.08 g/L) of polysaccharides were detected in permeate samples from the UF of red wine, whereas permeate from the UF of white wine contained marginally higher polysaccharide concentrations (i.e., 0.07–0.16 g/L). The propensity for polysaccharides to bind with other wine macromolecules, especially phenolic compounds, may explain their near absence in permeate. Again, from a wine sensory perspective, polyphenolics (including tannins) and polysaccharides are known to contribute desirable textural properties [44]. As a consequence, their removal (alongside that of anthocyanins) saw permeate stripped of the essential characters of red wine, such that they were no longer considered to be commercially acceptable as red wine, but again, the permeate could be used in white or rose wine, or as a blending component.

### 3.3. Remediation of a Highly Phenolic Wine by UF

The addition of grape marc to white wine for a period of three months (with ullage) yielded an excessively phenolic and oxidised wine, as intended (Table 4). Significant increases in total phenolics and polysaccharides were achieved, i.e., 6.2- and 5.7-fold increases, respectively. The wine also exhibited visual signs of browning (Figure S1) and returned an A_420_ measure (0.17 a.u.) indicative of elevated brown pigments [42]. Wine pH and TA were also affected, whereby pH increased from 3.03 to 4.0, while TA decreased from 6.1 to 5.0 g/L. This was attributed to the precipitation of potassium bitartrate, facilitated by the extraction of additional potassium and tartaric acid from the grape marc, and prolonged storage at low temperature (10 °C), which would have decreased its solubility, and therefore, tartrate stability [42]. A negligible (0.1%) decrease in alcohol content was observed.

The potential for UF to remediate excessive phenolic compounds and brown pigments from white wine was demonstrated by comparing the composition of retentate and permeate following 95% fractionation of the high-phenolic wine using the 10 kDa MWCO membrane (Table 4).

A ~50% reduction in the phenolic content of permeate was achieved by UF, while the concentration of brown pigments decreased by ~70%, and polysaccharides were not detected in the permeate at all. These macromolecules were concentrated in the resulting retentate; however, mass balance calculations accounted for only 75, 27, and 63% of phenolic compounds, polysaccharides, and brown pigments, respectively, relative to the concentrations observed in the phenolic wine prior to UF; Table S3). This suggests that in addition to macromolecules being concentrated, these compounds were also partially removed, either through adsorption to the membrane surface or solute binding, similar to that observed during the UF treatment of the white wine (Section 3.1). As a consequence, UF treatment gave permeate with significantly improved colour (diminished browning, Figure S1) and decreased phenolic compounds.

Small (≤0.2% abv) differences in alcohol content were observed amongst the retentate and permeate samples prepared from the high-phenolic wine, whereas pH was not affected. Acids were again retained by the UF membrane, resulting in an increase in retentate TA, with 90% of TA accounted for by the mass balance calculations (Table S3). This suggests that adsorption of organic acids by the membrane and/or solutes occurred to a greater extent than was observed during UF treatment of the original white wine; the loss of acids may have been exacerbated by the higher phenolic, polysaccharide, and bitartrate loads.

While the levels of phenolic extraction and browning achieved in the high-phenolic wine were extreme relative to what might be expected to occur during commercial white wine production, this study was necessary to explore potential winemaking applications of UF. The use of UF to mitigate the perception of negative sensory attributes arising from excess wine phenolics has already been demonstrated at commercial scale [27], but results from the current study suggest that UF could be exploited in other ways. For example, UF could address issues of discolouration; not only browning due to oxidation, but also pinking [45], without the need for the addition of fining agents, which are not always selective and can therefore also remove desirable wine constituents [19]. More importantly, UF could be used to remove the excess levels of phenolic compounds that arise in wines made from heavily pressed juice fractions, thereby improving juice recovery, quality, and profitability, without the need for the use of fining agents; some of which winemakers are looking to phase out. In this way, winemakers could increase the production volume of higher quality wine, by transforming wine derived from heavily pressed juice fractions into a more acceptable blending component. The potential uses of retentate should also be explored. For example, the phenolic, polysaccharide, and protein-rich retentate derived from UF of wine could be used to modify the sensory profiles of other wines; retentate could be used to help stabilise colour in pinot noir fermentations, or to address the textural deficiencies inherent in dealcoholised (no- and low-alcohol) wines.

## 4. Conclusions

Results from this study have demonstrated the partitioning of key wine constituents by UF, using membranes with different nominal MWCO specifications. Fractionation of organic acids and alcohol largely followed the degree of permeation, although partial loss of acids was observed, especially following UF of red wine beyond 90% permeation using a 10 kDa membrane. In contrast, a significant retention (and also removal) of key macromolecules was observed, irrespective of membrane MWCO. As expected, UF stripped red wine permeates of much of their essential character, namely colour and mouthfeel, so UF may have limited practical applications in red wine production. It is also not commercially viable to routinely discard ≥10% of wine volume, so UF treatment of white wines would need to achieve ≥90% permeation, and/or beneficial uses for retentate need to be identified and demonstrated. Nevertheless, the current study enabled partitioning of wine constituents by membranes of different MWCO to be studied under replicated experimental conditions. It is important to note, however, that the compositional outcomes of UF may vary by wine and by treatment, because factors such as molecule concentration and shape, as well as operating conditions (temperature, pressure, and flow rate) affect separation efficiency. Furthermore, the retention characteristics of membranes, even those with the same nominal MWCO specifications, will vary, especially given that methods for determining MWCO are not always comparable (i.e., different membrane manufacturers use different standards). The current study also demonstrated the use of UF to remediate a high-phenolic wine with obvious brown colouration. This is particularly important given the phasing out of fining agents traditionally used in white winemaking. A range of additional winemaking applications were also proposed, from the transformation of wine derived from heavily pressed juice fractions into commercially acceptable blending components, to the use of retentate to enhance the colour stability of red wine or to overcome the mouthfeel deficiencies of dealcoholised wine—and are now the subject of ongoing research.

## Figures and Tables

**Table 1:** Fractionation endpoints for pilot-scale UF treatment of white and red wines.

Degree of Permeation	Feed Volume (mL)	Permeate Volume (mL)	Retentate Volume (mL)
50%	5	2.5	2.5
80%	5	4	1
90%	10	9	1
95%	20	19	1

**Table 2:** Physico-chemical composition of white wine, and retentate and permeate samples obtained from pilot-scale ultrafiltration treatment with 20 and 10 kDa membranes and different degrees of permeation.

	Membrane MWCO (kDa)	Permeation Degree (%)	pH *	TA (g/L) *	Conductivity (ms/cm) *	Alcohol (abv) *	Protein (mg/L) *	Total Phenolics (a.u.) *	Polysaccharides (g/L) *
Wine	–	–	3.03 ± 0.01 e	6.1 ± 0.01 e	1.62 ± 0.001 f	11.9 ± 0.01 ab	17.0 ± 0.1 f	2.10 ± 0.01 e	0.29 ± 0.001 e
Retentate	20	50	3.23 ± 0.01 ab	6.2 ± 0.01 e	1.72 ± 0.003 e	11.7 ± 0.01 c	13.5 ± 0.2 h	1.54 ± 0.05 f	0.29 ± 0.010 e
		80	3.23 ± 0.01 ab	6.5 ± 0.03 d	1.81 ± 0.001 cd	11.9 ± 0.01 b	15.3 ± 0.1 g	2.59 ± 0.01 d	0.57 ± 0.012 d
		90	3.24 ± 0.01 a	6.7 ± 0.01 c	1.80 ± 0.003 d	12.0 ± 0.01 ab	27.7 ± 0.3 e	3.28 ± 0.04 c	0.89 ± 0.027 c
		95	3.21 ± 0.01 c	7.3 ± 0.01 b	1.88 ± 0.001 b	12.1 ± 0.12 a	61.4 ± 0.5 b	6.09 ± 0.13 b	1.82 ± 0.204 b
	10	50	3.23 ± 0.01 abc	5.7 ± 0.03 f	1.70 ± 0.020 e	11.0 ± 0.01 e	15.2 ± 0.1 g	1.89 ± 0.01 e	0.30 ± 0.009 e
		80	3.22 ± 0.01 bc	6.3 ± 0.15 e	1.79 ± 0.003 d	11.3 ± 0.02 d	37.0 ± 0.1 d	2.79 ± 0.01 d	0.57 ± 0.028 d
		90	3.22 ± 0.01 abc	6.3 ± 0.05 e	1.84 ± 0.005 c	11.2 ± 0.01 d	56.2 ± 0.8 c	3.48 ± 0.04 c	1.60 ± 0.078 b
		95	3.19 ± 0.01 d	7.7 ± 0.03 a	2.00 ± 0.001 a	12.0 ± 0.01 ab	170.4 ± 0.1 a	8.59 ± 0.02 a	2.20 ± 0.082 a
	*p*		<0.0001	<0.0001	<0.0001	<0.0001	<0.0001	<0.0001	<0.001
Wine	–	–	3.03 ± 0.01 e	6.1 ± 0.01 a	1.62 ± 0.001 cd	11.9 ± 0.01 a	17.0 ± 0.1 a	2.10 ± 0.01 a	0.29 ± 0.001 a
Permeate	20	50	3.25 ± 0.01 b	5.6 ± 0.02 cd	1.59 ± 0.001 ab	11.3 ± 0.02 e	6.4 ± 0.3 b	1.49 ± 0.01 d	0.13 ± 0.015 bc
		80	3.26 ± 0.01 a	5.7 ± 0.04 bc	1.63 ± 0.002 bc	11.6 ± 0.01 d	6.4 ± 0.4 b	1.61 ± 0.01 c	0.12 ± 0.018 bcd
		90	3.26 ± 0.01 a	5.8 ± 0.05 bc	1.64 ± 0.001 ab	11.6 ± 0.01 c	5.1 ± 0.1 b	1.62 ± 0.01 c	0.12 ± 0.010 bcd
		95	3.26 ± 0.01 a	5.8 ± 0.11 bc	1.65 ± 0.002 a	11.8 ± 0.02 b	5.9 ± 0.1 b	1.89 ± 0.01 b	0.16 ± 0.030 b
	10	50	3.22 ± 0.01 d	5.0 ± 0.09 e	1.56 ± 0.004 f	10.0 ± 0.01 h	5.2 ± 1.1 b	0.20 ± 0.01 g	0.08 ± 0.005 de
		80	3.25 ± 0.01 b	5.5 ± 0.06 d	1.60 ± 0.0105 e	10.2 ± 0.02 g	5.4 ± 0.1 b	0.61 ± 0.01 f	0.08 ± 0.020 de
		90	3.24 ± 0.01 c	5.5 ± 0.04 d	1.62 ± 0.006 d	11.0 ± 0.01 f	5.1 ± 0.2 b	0.79 ± 0.01 e	0.07 ± 0.004 e
		95	3.25 ± 0.01 b	5.9 ± 0.01 ab	1.64 ± 0.001 a	11.7 ± 0.04 c	6.0 ± 0.3 b	1.63 ± 0.04 c	0.10 ± 0.018 cde
	*p*		<0.0001	<0.0001	<0.0001	<0.0001	<0.0001	<0.0001	<0.001

Values are means of experimental duplicates (*n* = 2) ± standard deviation. Different letters (within columns, for retentate and permeate) indicate statistical significance (one-way ANOVA, Tukey’s HSD post-hoc, *p* = 0.05); * denotes significant differences between corresponding retentate and permeate samples (following >50% permeation; two-way ANOVA, Tukey’s HSD post-hoc, *p* = 0.05).

**Table 3:** Physico-chemical composition of red wine, and retentate and permeate samples, obtained from pilot-scale ultrafiltration treatment with 75, 20, and 10 kDa membranes and different degrees of permeation.

	Membrane MWCO (kDa)	Permeation Degree (%)	pH	TA (g/L) *	Conductivity (ms/cm)	Alcohol (abv)	Total Anthocyanins (mg/L) *	Colour Density (a.u.) *	Total Phenolics (a.u.) *	Tannins/ Epicatechin (g/L) *	Polysaccharides (g/L) *
Wine	–	–	3.52 ± 0.01 ab	6.7 ± 0.07 f	2.64 ± 0.01 f	14.7 ± 0.01 a	453 ± 2.9 g	13.7 ± 0.03 g	56 ± 0.71 f	1.09 ± 0.01 e	1.11 ± 0.03 f
Retentate	75	50	3.56 ± 0.01 a	7.1 ± 0.01 ef	2.65 ± 0.01 f	14.2 ± 0.35 bcd	633 ± 25 fg	19.0 ± 0.01 f g	57 ± 7.4 f	1.27 ± 0.01 e	1.00 ± 0.08 f
		80	3.49 ± 0.02 b	8.0 ± 0.14 de	2.82 ± 0.06 def	14.2 ± 0.07 bcd	73 ± 88 de	37.0 ± 0.59 e	86 ± 4.4 e	2.34 ± 0.21 d	1.80 ± 0.29 def
		90	3.50 ± 0.01 b	8.7 ± 0.49 cd	2.91 ± 0.08 cde	14.3 ± 0.28 bcd	1175 ± 19 d	43.7 ± 0.11 de	122 ± 9.7 cd	2.58 ± 0.13 cd	2.17 ± 0.37 de
		95	3.49 ± 0.01 b	10.8 ± 0.21 c	3.08 ± 0.01 bc	14.2 ± 0.01 bcd	2006 ± 1.0 ab	62.5 ± 0.77 b	190 ± 5.6 b	3.27 ± 0.09 b	2.75 ± 0.10 d
	20	50	3.52 ± 0.01 ab	7.2 ± 0.01 ef	2.62 ± 0.04 f	14.2 ± 0.01 bcd	698 ± 18 fg	20.4 ± 0.06 fg	63 ± 5.9 f	1.38 ± 0.02 e	1.17 ± 0.11 ef
		80	3.50 ± 0.01 b	8.5 ± 0.07 d	2.72 ± 0.23 ef	14.3 ± 0.01 bcd	1194 ± 136 d	40.0 ± 0.65 de	113 ± 14.4 d	2.68 ± 0.03 c	2.27 ± 0.15 d
		90	3.50 ± 0.03 b	8.8 ± 0.28 cd	2.83 ± 0.02 def	14.5 ± 0.01 abc	1868 ± 179 bc	49.4 ± 0.59 cd	135 ± 9.6 c	3.09 ± 0.11 b	5.60 ± 0.06 c
		95	3.49 ± 0.01 b	9.6 ± 1.3 c	2.97 ± 0.08 bcd	14.6 ± 0.07 ab	2209 ± 233 a	74.3 ± 0.80 a	212 ± 8.5 a	3.97 ± 0.01 a	13.28 ± 0.34 b
	10	50	3.50 ± 0.03 d	9.1 ± 0.14 c	3.16 ± 0.03 b	14.0 ± 0.07 d	818 ± 35 ef	26.2 ± 0.41 f	73 ± 9.3 ef	1.37 ± 0.02 e	1.29 ± 0.06 ef
		80	3.42 ± 0.04 c *	12.5 ± 0.14 b	3.98 ± 0.21 a	13.5 ± 0.14 e	1700 ± 87 ac	50.3 ± 0.03 cd	166 ± 10.0 b	3.25 ± 0.09 b	2.87 ± 0.48 d
		90	3.42 ± 0.01 c *	13.5 ± 0.42 a	3.91 ± 0.03 a	14.1 ± 0.01 cd	1952 ± 201 bc	55.4 ± 0.09 bc	166 ± 10.7 b	3.72 ± 0.06 a	15.65 ± 0.71 a
	*p*		0.012	<0.0001	<0.0001	<0.0001	<0.0001	<0.0001	<0.0001	<0.0001	<0.0001
Wine	–	–	3.52 ± 0.01 d	6.7 ± 0.07 a	2.64 ± 0.01 a	14.7 ± 0.01 a	453 ± 2.9 a	13.7 ± 0.03 a	56 ± 0.71 a	1.09 ± 0.01	1.11 ± 0.03 a
Permeate	75	50	3.53 ± 0.01 d	5.8 ± 0.14 abc	2.34 ± 0.08 b	14.1 ± 0.14 c	152 ± 43 b	3.8 ± 0.13 b	19.4 ± 4.60 b	nd	0.08 ± 0.01 b
		80	3.53 ± 0.01 d	5.9 ± 0.14 abc	2.39 ± 0.06 b	14.1 ± 0.07 c	150 ± 23 b	3.6 ± 0.04 b	19.5 ± 2.69 b	nd	0.03 ± 0.01 b
		90	3.53 ± 0.01 d	6.2 ± 0.07 abc	2.42 ± 0.01 b	14.5 ± 0.07 b	167 ± 22 b	4.2 ± 0.08 b	20.9 ± 2.47 b	nd	0.08 ± 0.01 b
		95	3.53 ± 0.01 d	6.2 ± 0.07 abc	2.40 ± 0.01 b	14.6 ± 0.07 ab	139 ± 9 b	3.3 ± 0.02 b	18.0 ± 0.71 b	nd	0.03 ± 0.01 b
	20	50	3.53 ± 0.01 d	5.4 ± 0.07 cd	2.30 ± 0.03 b	13.8 ± 0.01 d	124 ± 52 b	2.9 ± 0.13 b	16.2 ± 5.0 b	nd	0.04 ± 0.05 b
		80	3.62 ± 0.13 a	5.5 ± 0.21 bcd	2.36 ± 0.03 b	14.1 ± 0.01 c	112 ± 14 c	2.4 ± 0.02 b	15.0 ± 0.9 b	nd	0.04 ± 0.01 b
		90	3.52 ± 0.02 d	6.0 ± 0.07 abc	2.36 ± 0.06 b	14.4 ± 0.01 b	122 ± 25 b	2.6 ± 0.04 b	16.5 ± 2.6 b	nd	0.05 ± 0.01 b
		95	3.52 ± 0.04 d	6.3 ± 0.57 ab	2.48 ± 0.09 ab	14.5 ± 0.01 b	161 ± 41 b	3.4 ± 0.09 b	20.6 ± 4.4 b	nd	0.05 ± 0.01 b
	10	50	3.61 ± 0.01 ab	4.6 ± 0.42 d	1.75 ± 0.01 c	13.9 ± 0.01 d	11.4 ± 2.4 c	0.3 ± 0.01 c	2.50 ± 0.14 c	nd	0.05 ± 0.07 b
		80	3.58 ± 0.01 c *	4.7 ± 0.42 d	1.82 ± 0.01 c	14.1 ± 0.07 c	10.3 ± 1.4 c	0.3 ± 0.01 c	2.70 ± 0.57 c	nd	0.04 ± 0.06 b
		90	3.59 ± 0.01 bc *	4.7 ± 0.07 d	1.75 ± 0.01 c	14.4 ± 0.14 b	7.4 ± 0.9 c	0.2 ± 0.01 c	2.60 ± 0.57 c	nd	n.d.
	*p*		<0.001	<0.0001	<0.0001	<0.0001	<0.0001	<0.0001	<0.0001	na	<0.0001

Values are means of experimental duplicates (*n* = 2) ± standard deviation. Different letters (within columns, for retentate and permeate) indicate statistical significance (one-way ANOVA, Tukey’s HSD post-hoc, *p* = 0.05); nd = not detected; na = not available; * denotes significant differences between corresponding retentate and permeate samples (following >50% permeation; two-way ANOVA, Tukey’s HSD post-hoc, *p* = 0.05).

**Table 4:** Physico-chemical composition of the phenolic wine, and retentate and permeate samples obtained from pilot-scale ultrafiltration treatment with a 10 kDa membrane and 95% permeation.

	pH	TA (g/L)	Alcohol (abv)	Total Phenolics (a.u.)	Polysaccharides (g/L)	Brown Pigments (a.u.)
Base Wine	3.03 ± 0.01	6.1 ± 0.01	11.9 ± 0.01	2.10 ± 0.01	0.29 ± 0.001	–
Phenolic Wine	4.0 ± 0.01	5.0 ± 0.1 b	11.8 ± 0.02 a	13.1 ± 0.6 b	1.64 ± 0.05 b	0.17 ± 0.01 b
Retentate	4.0 ± 0.01	8.0 ± 0.2 a	11.7 ± 0.02 a	71.9 ± 2.0 a	8.93 ± 0.72 a	1.20 ± 0.01 a
Permeate	4.0 ± 0.01	4.3 ± 0.5 b	11.6 ± 0.11 b	6.6 ± 0.2 c	nd	0.05 ± 0.01 c
*p*	ns	<0.001	0.008	<0.001	<0.001	<0.001

Values are means of experimental duplicates (*n* = 2) ± standard deviation for the base wine and means of experimental triplicates (*n* = 3) ± standard deviation for other samples; n.d. = not detected. Different letters indicate statistical significance (one-way ANOVA, Tukey’s HSD post-hoc, *p* = 0.05) amongst phenolic wine, retentate, and permeate samples; nd = not detected; ns = not significant.

## Data Availability

The original contributions presented in the study are included in the article/Supplementary Materials, further inquiries can be directed to the corresponding author.

## Supplementary Materials

The following supporting information can be downloaded at: https://www.mdpi.com/article/10.3390/foods13121850/s1, Figure S1: Changes in wine colour observed following pilot-scale ultrafiltration treatment of (a) a red wine and (b) a high-phenolic wine; Table S1: Relative proportion of titratable acidity (TA), alcohol, protein, total phenolics and polysaccharides observed in retentate (R) and permeate (P), (and overall, ∆, relative to initial wine composition), following pilot-scale ultrafiltration of white wine with 20 and 10 kDa MWCO membranes and different degrees of permeation; Table S2: Relative proportion of titratable acidity (TA), alcohol, protein, total phenolics and polysaccharides observed in retentate (R) and permeate (P), (and overall, ∆, relative to initial wine composition), following pilot-scale ultrafiltration of red wine with 75, 20 and 10 kDa MWCO membranes and different degrees of permeation; Table S3: Relative proportion of titratable acidity (TA), alcohol, total phenolics, polysaccharides and brown pigments observed in retentate (R) and permeate (P), (and overall, ∆, relative to initial wine composition), following pilot-scale ultrafiltration of an excessively phenolic wine with a 10 kDa MWCO membrane and 95% permeation.
