# Supplementary material for: Compositional Consequences of Ultrafiltration Treatment of White and Red Wines
Source: Foods. 2024 Jun 13;13(12):1850. doi: 10.3390/foods13121850 (PMC11202439; doi:10.3390/foods13121850)
Supplement: Supplementary file 1 [file foods-13-01850-s001.zip › foods-3023264-supplementary.pdf]

foods

MDPI

Supplementary Materials

Article

Compositional Consequences of Ultrafiltration Treatment of White and Red Wines

Stephanie Angela [1,2], David Wollan [2,3], Richard Muhlack [1,2], Keren Bindon [4], and Kerry Wilkinson [1,2,*]

[1] Discipline of Wine Science and Waite Research Institute, The University of Adelaide, PMB 1, Glen Osmond, SA 5064, Australia; stephanie.angela@adelaide.edu.au; richard.muhlack@adelaide.edu.au;
[2] The Australian Research Council Training Centre for Innovative Wine Production, PMB 1, Glen Osmond, SA 5064, Australia;
[3] VAF Memstar, PO Box 794, Nuriootpa, SA 5355, Australia; david.wollan@gmail.com;
[4] The Australian Wine Research Institute, PO Box 197, Glen Osmond, SA 5064, Australia; keren.bindon@awri.com.au

* Correspondence: kerry.wilkinson@adelaide.edu.au

Table S1. Relative proportion of titratable acidity (TA), alcohol, protein, total phenolics and polysaccharides observed in retentate (R) and permeate (P), (and overall, Δ, relative to initial wine composition), following pilot-scale ultrafiltration of white wine with 20 and 10 kDa MWCO membranes and different degrees of permeation.

**Membrane MWCO (kDa)**	**Permeation Degree (%)**	**TA (g/L)**			**Alcohol (abv)**			**Protein (mg/L)**			**Total Phenolics (a.u.)**			**Polysaccharides (g/L)**		
		R	P	Δ	R	P	Δ	R	P	Δ	R	P	Δ	R	P	Δ
20	50	51%	46%	**97%**	49%	47%	**97%**	50%	19%	**59%**	37%	35%	**72%**	50%	22%	**72%**
	80	21%	75%	**96%**	20%	78%	**98%**	18%	30%	**48%**	25%	61%	**86%**	39%	33%	**72%**
	90	11%	86%	**97%**	10%	88%	**98%**	16%	27%	**43%**	16%	69%	**85%**	31%	37%	**68%**
	95	6%	90%	**96%**	5%	94%	**99%**	18%	33%	**51%**	15%	85%	**100%**	31%	52%	**84%**
10	50	47%	41%	**88%**	46%	42%	**88%**	45%	15%	**60%**	45%	5%	**50%**	52%	14%	**66%**
	80	21%	72%	**93%**	19%	69%	**88%**	44%	25%	**69%**	27%	23%	**50%**	39%	22%	**61%**
	90	10%	81%	**91%**	9%	83%	**93%**	33%	27%	**60%**	17%	34%	**50%**	55%	22%	**77%**
	95	6%	92%	**98%**	5%	93%	**98%**	50%	34%	**84%**	20%	74%	**94%**	38%	33%	**71%**

Table S2. Relative proportion of titratable acidity (TA), alcohol, protein, total phenolics and polysaccharides observed in retentate (R) and permeate (P), (and overall, Δ, relative to initial wine composition), following pilot-scale ultrafiltration of red wine with 75, 20 and 10 kDa MWCO membranes and different degrees of permeation.

Membrane MWCO (kDa)	Permeation Degree (%)	TA (g/L)			Alcohol (abv)			Total Anthocyanins (mg/L)			Total Phenolics (a.u.)			Polysaccharides (g/L)		
		R	P	Δ	R	P	Δ	R	P	Δ	R	P	Δ	R	P	Δ
75	50	53%	43%	**96%**	48%	48%	**96%**	70%	17%	**87%**	51%	17%	**68%**	45%	4%	**49%**
	80	24%	70%	**94%**	19%	77%	**96%**	43%	26%	**69%**	31%	28%	**59%**	32%	2%	**35%**
	90	13%	83%	**96%**	10%	89%	**99%**	26%	33%	**59%**	22%	34%	**55%**	20%	6%	**26%**
	95	8%	88%	**96%**	5%	94%	**99%**	22%	29%	**51%**	17%	31%	**48%**	12%	3%	**15%**
20	50	54%	40%	**94%**	48%	47%	**95%**	77%	14%	**91%**	56%	14%	**71%**	53%	2%	**55%**
	80	25%	66%	**91%**	19%	77%	**96%**	53%	20%	**72%**	40%	21%	**62%**	41%	3%	**44%**
	90	13%	81%	**94%**	10%	88%	**98%**	41%	24%	**65%**	24%	27%	**51%**	50%	4%	**55%**
	95	7%	89%	**96%**	5%	94%	**99%**	24%	34%	**58%**	19%	35%	**54%**	60%	4%	**64%**
10	50	68%	34%	**102%**	48%	47%	**95%**	90%	1%	**92%**	65%	2%	**67%**	58%	2%	**60%**
	80	37%	56%	**93%**	18%	77%	**95%**	75%	2%	**77%**	59%	4%	**63%**	52%	3%	**55%**
	90	20%	63%	**83%**	10%	88%	**98%**	43%	1%	**45%**	30%	4%	**34%**	141%	0%	**141%**

Table S3. Relative proportion of titratable acidity (TA), alcohol, total phenolics, polysaccharides and brown pigments observed in retentate (R) and permeate (P), (and overall, Δ, relative to initial wine composition), following pilot-scale ultrafiltration of an excessively phenolic wine with a 10 kDa MWCO membrane and 95% permeation.

**TA (g/L)**			**Alcohol (abv)**			**Total Phenolics (a.u.)**			**Polysaccharides (g/L)**			**Brown Pigments (a.u.)**		
R	P	Δ	R	P	Δ	R	P	Δ	R	P	Δ	R	P	Δ
8%	82%	**90%**	5%	93%	**98%**	27%	48%	**75%**	27%	0%	**27%**	35%	28%	**63%**

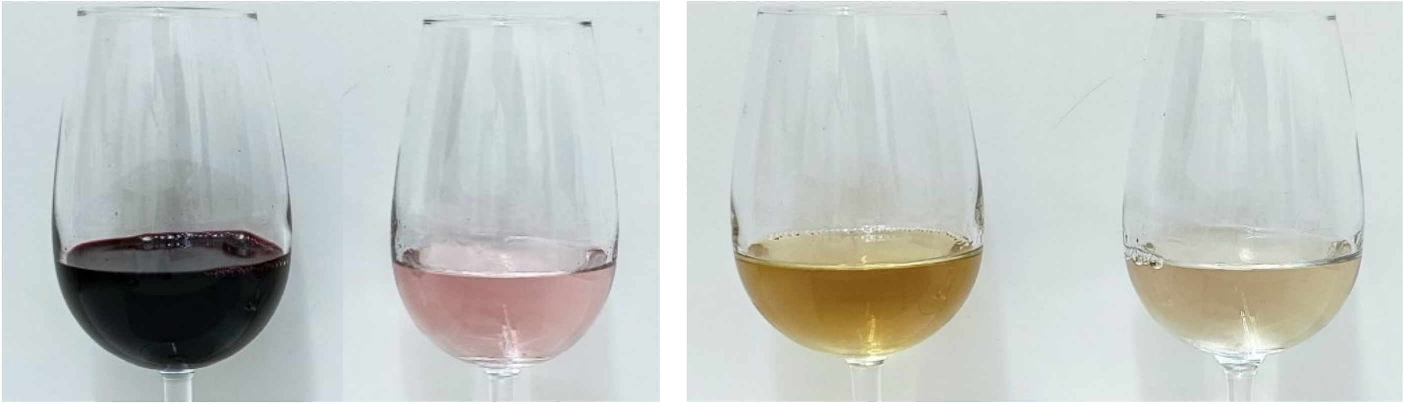

(a) **(b)**

Figure S1. Changes in wine colour observed following pilot-scale ultrafiltration treatment of **(a)** a red wine and **(b)** a high-phenolic wine.
